# Effect of Goji Berry (*Lycium barbarum*) Supplementation on Reproductive Performance of Rabbit Does

**DOI:** 10.3390/ani11061672

**Published:** 2021-06-03

**Authors:** Egon Andoni, Giulio Curone, Stella Agradi, Olimpia Barbato, Laura Menchetti, Daniele Vigo, Riccardo Zelli, Elisa Cotozzolo, Maria Rachele Ceccarini, Massimo Faustini, Alda Quattrone, Marta Castrica, Gabriele Brecchia

**Affiliations:** 1 Faculty of Veterinary Medicine, Agricultural University of Albania, 1029 Kamez, Albania; eandoni@ubt.edu.al; 2 Department of Veterinary Medicine, University of Milano, 26900 Lodi, Italy; giulio.curone@unimi.it (G.C.); stella.agradi@unimi.it (S.A.); daniele.vigo@unimi.it (D.V.); massimo.faustini@unimi.it (M.F.); gabriele.brecchia@unimi.it (G.B.); 3 Department of Veterinary Medicine, University of Perugia, 06121 Perugia, Italy; olimpia.barbato@unipg.it (O.B.); riccardo.zelli@unipg.it (R.Z.); alda.quattrone@hotmail.it (A.Q.); 4 Department of Agricultural and Food Sciences, University of Bologna, 40137 Bologna, Italy; 5 Department of Agricultural, Food and Environmental Sciences, University of Perugia, 06121 Perugia, Italy; elisa.cotozzolo@studenti.unipg.it; 6 Department of Pharmaceutical Sciences, University of Perugia, 06123 Perugia, Italy; mariarachele.ceccarini@unipg.it; 7 Department of Health, Animal Science and Food Safety “Carlo Cantoni”, University of Milano, 20133 Milan, Italy; marta.castrica@unimi.it

**Keywords:** receptivity, fertility, estrogen, LH, milk production

## Abstract

**Simple Summary:**

Infectious diseases represent serious problems for the reproductive performance of livestock animals because they negatively affect not only the welfare of the animals, but also the profitability of the farm. Moreover, the European Community continues to promote the reduction of the use of antibiotics and hormones in animal breeding. In this context, it is necessary to find new nutritional approaches to reduce the negative energy balance, and at the same time, to reinforce the immune system of the animals. In this research, the effect of goji berry supplementation on the reproductive activity and productive performance of rabbits is evaluated. *Lycium barbarum* fruit is considered a nutraceutical natural product containing various biologically active substances that show health benefits for both humans and animals. In particular, the berry can modulate hormones and metabolites involved in energy balance and reproduction, stimulate and balance the immune system activity, contributing to the defense of the organism against pathogens. Our results suggest that the integration with goji berry in the rabbit diet at 1% affects the reproductive activity, influencing the pattern secretion of luteinizing hormone (LH) and estrogens, as well as the sexual receptivity. Moreover, the fruit induced a higher milk production, improving the productive performance of young rabbits.

**Abstract:**

Goji berry shows a wide range of beneficial properties in human health, but only a few studies evaluated its effects in livestock animals. The objective of this research was to assess the effects of goji berry supplementation on the hormonal profile, productive, and reproductive performance of does. Two months before artificial insemination, 105 nulliparous does were randomly divided into three groups (*n* = 35) based on the dietary treatment: commercial diet (C), or a diet supplemented with either 1% (G1), or 3% (G3) of goji berry, respectively. The results showed that receptivity was higher in G1 than in the C group (*p* < 0.05). Trends toward significance for differences between the G1 and G3 groups in marginal means of LH concentrations (*p* = 0.059), and between G1 and C in LH AUC values (*p* = 0.078), were evidenced. Estrogen concentrations showed a more fluctuating trend but a significant interaction effect (*p* < 0.001). The G1 group showed higher litter weight than C at birth (*p* = 0.008) and weaning (*p* < 0.001), as well as higher litter size at weaning (*p* = 0.020). The G1 group also exhibited the highest mean milk production (*p* < 0.01). In conclusion, goji berry influenced reproductive and productive performance, probably via modulating hormonal patterns and milk production in rabbits. However, further studies are needed to validate these preliminary results.

## 1. Introduction

*Lycium barbarum*, also known as wolfberry or Goji berry, is a functional food and plant medicine that has been used in China and Asian countries for 2300 years to restore well-being, improve eyesight, and nourish the kidneys and liver [1]. Recent studies have shown that goji berries possess various benefits for human health such as anti-aging [2], antioxidant [3], antidiabetic [4], hypolipidaemic [5], anticancer [6], cytoprotective [7], neuroprotective [8,9], immunomodulatory [10], gastroprotective [11], radiation protecting [12], and visual protecting effects [13]. As a consequence of all these beneficial properties and of the increasing public awareness of health and the quality of life, the plant and the fruit of *Lycium barbarum* have become extraordinarily popular in Western countries, where its cultivation and consumption have increased [1]. Moreover, the fruit seems to have a high safety profile at different dosages [14], although a few studies reported mild toxicity [15] and adverse effects such as urticarial-like reactions related to its use [16]. Goji berry contains a high quantity of carbohydrates, dietary fiber, protein, macro and micronutrients, and low levels of fat [17]. Besides the high nutritional value, it contains many biologically active compounds such as polysaccharides, carotenoids, phenylpropanoids, phenolics, and flavonoids [18]. Several studies showed that the wide range of effects of goji berries are principally due to the biological properties of polysaccharides [1]. They represent the most abundant constituents of the fresh and dried berry (46–51% and 5–8%, respectively), and are found principally branched and in the water-soluble form [14]. 

The research activity concerning goji berries was mainly carried out on humans, laboratory animals, and on specific cell lines in vitro [4,9,10,19], while only a few investigations have been carried out on livestock animals [20], including rabbits [21,22,23]. The rabbit is considered a livestock animal, and the productive efficiency of rabbit farms is strongly influenced by reproductive performance, especially those of the rabbit does. Generally, nulliparous does show higher fertility than primiparous and multiparous does [24,25]. The major causes of the reduced fertility rate of primiparous does are both the intensive reproductive rhythms to which they are subjected, and the negative energy balance due to the overlap between pregnancy and lactation [26,27]. Moreover, the profitability of the breeders could be reduced by the high culling and mortality rate of the animals, and the costs related to the purchase of medicines and veterinary services as a consequence of the sanitary status of the farm. Poor hygiene and incorrect artificial insemination practices are often linked to the onset of clinical or subclinical endometritis and metritis, which reduce the reproductive performance of the does [28,29]. Local and systemic diseases and/or inflammatory status induce the release of chemokines by the activation of TLR4 receptors that mobilize and activate immune cells [30]. At the systemic level, an alteration of the hormonal secretion of the gonadal axis could be induced; whereas at a local level, the inflammatory mediators and other signaling molecules can influence cellular, vascular, and endocrine functions impairing the reproductive activity of the animals [31,32]. 

Infectious diseases are generally fought with antibiotics, yet in the last decade, the abuse of these drugs led to the onset of antibiotic resistance with a dangerous and direct impact on human and animal health [33,34]. For this reason, the European Community guidelines tend to reduce the use of antibiotics and hormones in animal husbandry, including rabbit farming [35]. In this context, there has been a growing interest in the study and research of nutraceutical products that show health-promoting effects and with a potential in the prevention and treatment of several human and animal diseases, including those of the reproductive system [36,37]. Although there are numerous studies on the biological activity of goji berries, their effects on the reproductive functions, gonadic axis activity, and fertility, are poorly known [38,39]. In particular, the research is strongly limited for the female of both humans and animals, including rabbit does [40,41]. The rabbit is, however, an excellent animal model for research, in particular with regards to the physiology of reproduction and several reproductive parameters [42,43,44]. 

Goji berry could be a natural strategy to improve the reproductive performance of rabbit farms. It is speculated that the fruit could reduce the incidence of reproductive infections/inflammation of the genital tract, acting principally on the immune system and the oxidative status of the organs as well as influencing the hormonal secretion of the ovarian axis. Therefore, the main objective of the present study is to evaluate the effect of goji berry dietary supplementation on the reproductive performance, fertility, LH, and estrogen secretion, as well as the milk production, of rabbit does.

## 2. Materials and Methods 

### 2.1. Animals and Experimental Design

The experiment was conducted at the farm of the Agricultural University of Tirana, Faculty of Veterinary Medicine, Albania. The animals were maintained in accordance with Legislative Decree No. 146, implementing Directive 98/58/EC regarding the protection of animals that were kept for farming purposes. The experimental protocol was run with the permission of the Ministry of Agriculture and Rural Development, National Authority of Veterinary and Plants protection (prot. 824/2020), of Albania. All efforts were made to minimize animal distress and to use only the number of animals necessary to produce reliable results. Moreover, the responsible veterinarian for the farm checked the rabbits for health and welfare states daily.

Nulliparous New Zealand White rabbits (*n* = 105) of 4 months of age, weighing 3.5–3.8 kg, were individually housed in controlled environmental conditions where the temperature ranged from +18 to +21 °C, the relative humidity from 55% to 65%, the artificial ventilation was 0.3 m^3^/s, and the lighting was scheduled 16 L:8 D at 40 lux. Rabbits were provided 150 g/d of commercial food and water ad libitum. The composition of the diet supplied to the does is described in Table 1 and is in agreement with previous studies [21,22,23]. Goji berries in dried form were provided by a farm of central Italy (Impresa Agricola Gianluca Bazzica, Foligno, Italy). They were ground into smaller pieces, mixed with the rest of the diet ingredients, and, finally, pelleted [23]. All rabbits completely consumed their daily rations.

The does were randomly divided into three different groups (*n* = 35/group), according to the dietary treatment: commercial diet (Control, C), and diet supplemented with 1% or 3% of goji berry, G1 and G3 groups, respectively. After a period of adaptation to the new feed for two months, the does were submitted to artificial insemination (AI) at 6 months of age, performed with a heterospermic pool of fresh semen (0.5 mL) diluted 1:5 in a commercial extender. At the moment of the insemination, receptivity was established by controlling for the color of the vulva, and ovulation was induced by an intramuscular injection of 10 μg of synthetic gonadotropin-releasing hormone (GnRH; Receptal, Hoechst-Roussel Vet, Milan, Italy) [31]. Day 0 was designed as the day of the insemination. Pregnancy was diagnosed by abdominal palpation 12 days after AI, and then, 25 pregnant rabbit does per group were followed until the weaning of the young rabbits (day 35). Lactation was controlled by opening the door of the nest one time a day until 18 days after parturition. 

On the day of the AI, blood samples were collected every 60 minutes, starting 120 minutes before and up to 240 minutes after the AI and GnRH injection to evaluate LH and 17-β estradiol concentrations. The samples were withdrawn from the marginal ear vein by a butterfly needle of 24G connected to a syringe of 2.5 mL. Blood samples were inserted into tubes containing EDTA, and immediately centrifuged at 3000× *g* for 15 min; furthermore, plasma was stored frozen until it was assayed for hormone levels. 

The following reproductive and productive indices were calculated: receptivity (color of the vulva [46] categorized as white, pink, or red), fertility (number of parturitions/number of inseminations × 100), milk production, litter weight (from delivery until day 18 of the whole litter, and from day 18 to weaning of the single animal), litter size, and pre-weaning mortality (calculated as the percentage of weaned kits/litter) [47]. From parturition to day 35, the mortality, the litter weight, and the litter size were recorded daily. Milk production was evaluated daily by weighing the does before and after suckling, from parturition until day 18 of lactation [48].

### 2.2. Hormone and Metabolite Assays

Plasma LH concentrations were evaluated using a commercial rabbit LH ELISA kit (Wuhan Fine Biotech Co., Ltd., Wuhan, Hubei, China). The determination procedure is based on a sandwich enzyme-linked immune-sorbent assay technology. The kit shows high sensitivity, with a limit of detection of 0.281 ng/mL and excellent specificity for the detection of LH. The intra- and inter-assay coefficients of variation were <8 and <10%, respectively. Values below and above the limits of detection of the test were considered to be 0 and 30 ng / mL, respectively.

Plasma 17β-oestradiol concentrations in plasma samples were assayed using a commercial RIA kit (Immunotech sro, Prague, Czech Republic) following the procedure indicated by the producer [49]. The limit of detection was 13,11 pg/ml and the intra- and inter-assay coefficients of variation were <14.4 and <14.5%, respectively. Values below the limit of detection of the test were considered to be 0 pg/mL.

### 2.3. Statistical Analysis

Kolmogorov-Smirnov and Levene tests were used to verify assumptions. Hormone concentrations and milk yield were analyzed by mixed-design ANOVA followed by multiple comparison tests corrected using the Bonferroni–Sidak method. Mixed design ANOVA evaluated the effect of dietary treatment (i.e., group effect; 3 levels: C, G1, and G3 groups), change over time (i.e., repeated-measures effect; 7 levels for hormone concentrations and 18 levels for milk yield), and their interaction. The LH AUC (area under the curve) was calculated for each animal by the trapezoid method using LH values at each sampling time point from 0 to 240 from the GnRH injection [48,50]. LH AUC, litter size, and weights were compared between groups by one-way ANOVA. Finally, receptivity and fertility were analyzed by Chi-square tests to evaluate if there was an association between these parameters and dietary treatment. The proportions of each group were then compared by pairwise z-tests. Statistical analyses were performed with SPSS Statistics version 25 (IBM, SPSS Inc., Chicago, IL, USA) and GraphPad Prism version 5.01 software (Inc., San Diego, CA, USA). We defined *p* ≤ 0.05 as significant and *p* < 0.1 as a trend.

## 3. Results

### 3.1. Hormone Concentrations

**Figure 1:**
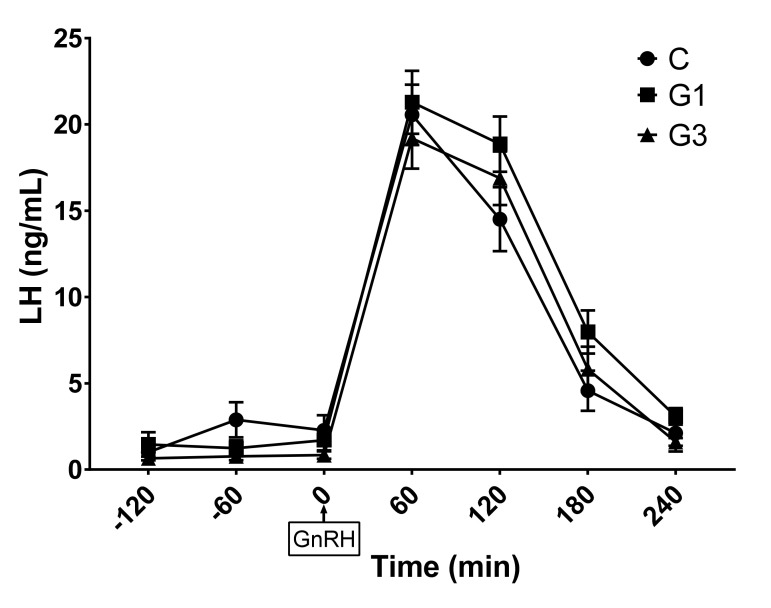
Plasma luteinizing hormone (LH) concentrations from minutes −120 to 240 after GnRH injection in the control group (C) and does supplemented with 1% (G1) or 3% (G3) of Goji. Values are means and standard errors.

Regardless of the group, plasma LH levels reached a peak at 60–120 minutes after GnRH injection (20.3 ± 1.1 ng/ml and 15.8 ± 2.2 ng/ml at 60 and 120 min, respectively; *p* for time effect <0.0001) and returned to baseline levels after 240 minutes (Figure 1). Regarding the group effect, trends toward significance were found for both LH concentrations (*p* = 0.056) and LH AUC (*p* = 0.067). In particular, multiple comparisons showed that marginal means of LH concentrations tended to be higher in G1 than in G3 (7.9 ± 3.2 and 6.5 ± 3.1 ng/ml for G1 and G3, respectively; *p* = 0.059), while LH AUC tended to be higher in G1 than in C (2510 ± 175 ng/ml x h and 3031 ± 149 ng/ml x h for C and G1, respectively; *p* = 0.078). 

**Figure 2:**
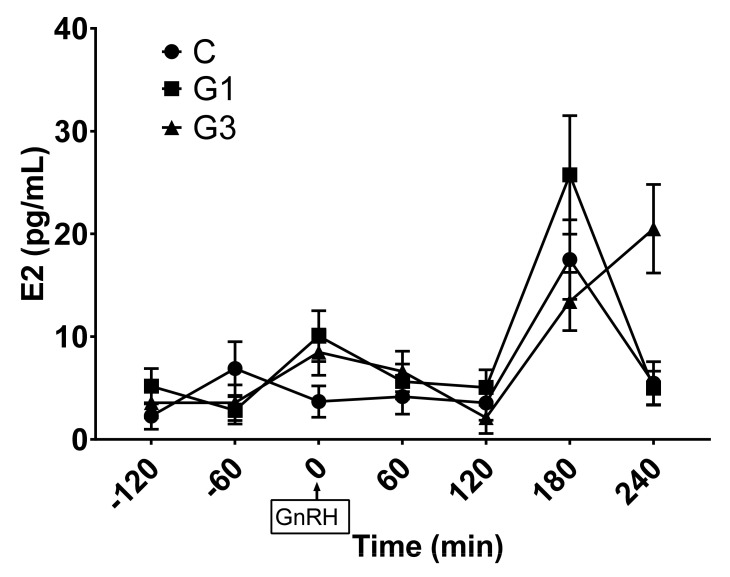
Plasma estrogen (E2) concentrations from minutes −120 to 240 after GnRH injection in the control group (C) and does supplemented with 1% (G1) or 3% (G3) of Goji. Values are means and standard errors.

Estrogen concentrations showed a more fluctuating trend, even if the highest mean values, regardless of group, were found at 180 minutes after GnRH injection (*p* for time effect <0.0001; Figure 2). Estrogen concentrations also showed a significant interaction effect (*p* < 0.001) and a trend toward significance for a group effect (*p* = 0.065). In particular, marginal means of the G1 group tended to be higher than in C (6.2 ± 1.9 pg/mL and 8.5 ± 3.0 pg/mL for C and G1, respectively; *p* = 0.088), and multiple comparisons showed significant differences between groups at time 0 (*p* = 0.034). Moreover, rabbits of the G3 group showed a delayed estrogen peak compared to the other two groups.

### 3.2. Reproductive and Productive Performance

**Figure 3:**
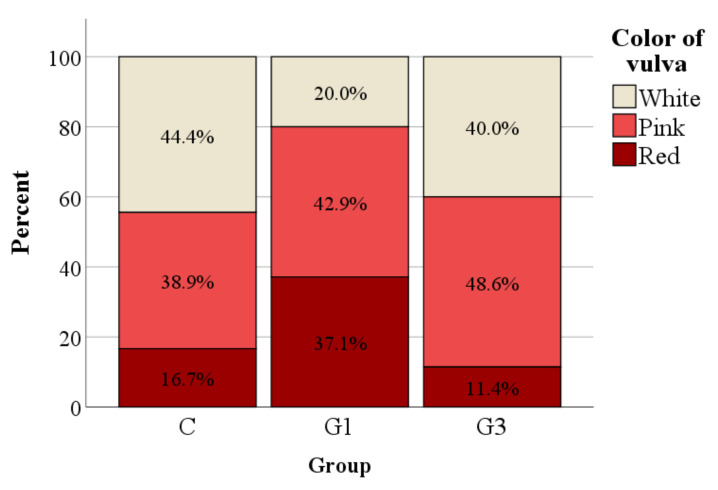
Relative frequency distribution of the color of the vulva used as an indicator of sexual receptivity in does of the control group (C) and does supplemented with 1% (G1) or 3% (G3) of Goji.

As an indicator of a rabbit doe’s receptivity, the color of the vulva was affected by nutritional treatment (*p* = 0.044). In particular, the percentage of does showing a white color of the vulva was lower in G1 than in the C group, while the percentage of does showing a red color was higher in G1 than in the G3 group (*p* < 0.05; Figure 3). Fertility, however, did not differ between groups (77%, 82%, and 74% for C, G1, and G3, respectively; *p* = 0.678). 

**Figure 4:**
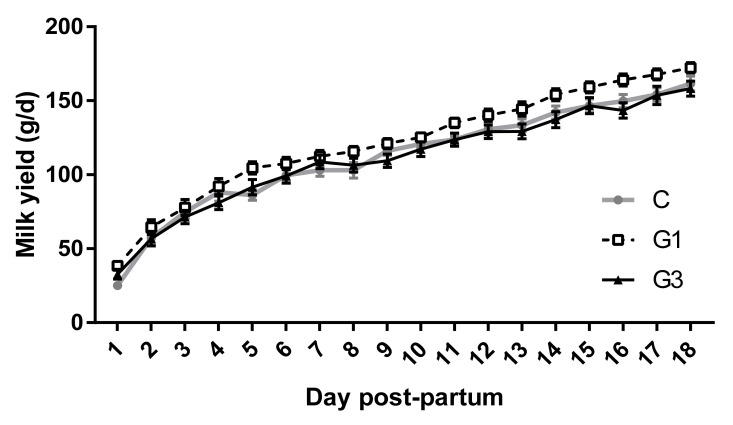
Milk production from day 1 to 18 post-partum of the control group (C) and does supplemented with 1% (G1) or 3% (G3) of Goji. Values are means and standard errors.

Milk yield increased from the 1st (32 ± 4 g/d) to the 18th day post-partum (164 ± 4 g/d; *p* < 0.001) in all groups (Figure 4), although, G1 showed the highest marginal means (112 ± 9 g/d, 122 ± 9 g/d, and 111 ± 8 g/d for the C, G1, and G3 groups, respectively; *p* < 0.05).

Pre-weaning mortality (*p* = 0.176) and litter size at birth (*p* = 0.249) did not differ between groups; however, rabbits of the G1 group showed higher litter size at weaning (*p* = 0.020), as well as higher litter weight at birth (*p* = 0.008) and at weaning (*p* < 0.001) compared to the C group. The G3 group showed intermediate values in litter weight at birth and litter size at weaning (*p* < 0.05), while their litter weight at weaning was higher than the C group (*p* < 0.01; Table 2).

## 4. Discussion

To our knowledge, this is the first study that evaluates the effect of goji berry on the reproductive activity of rabbit does. The present work suggests that the diet integration with goji berry could affect the hormonal pattern of LH and 17β-oestradiol, increase receptivity, milk production, litter weight at birth, litter weight, and size at weaning. The encouraging findings of our research indicate that the berry could be used in rabbit nutrition, although further studies are needed to confirm the current outcomes, in order to understand the mechanism of action as well as to evaluate the economic convenience of its use.

It has long been assumed that rabbit does do not have a well-defined estrus cycle, nor do they show well-specified estrus manifestation, so they are quite often erroneously considered to be in permanent estrus. Actually, the does present a period during which they accept mating (oestrus) as well as a period during which they reject the male (dioestrus). Sexual receptivity could be measured by a behavioral test in the presence of a male [51], and by evaluating the color and turgidity of the vulva [46]. In particular, the mating acceptance behavior in the presence of a male (lordosis position) and the red color of the vulva could be considered as estral manifestations of the doe. The relationships between sexual behavior, vulva color, and circulating concentrations of reproductive hormones have been studied at different reproductive stages, but a firm conclusion has not yet been reached [52,53]. Our results showed that, based on the evaluation of the color of the vulva, the sexual receptivity is higher in supplemented does, namely in the G1 group compared to the C group. Moreover, animals of the same group also showed higher 17β-oestradiol plasma concentrations compared to the other groups at the moment of AI, suggesting a link with the receptivity. In agreement with our findings, the results of several studies highlight that the turgidity and the red color of the vulva, and more in general, the receptive behavior of the doe, can be related to high plasma levels of estradiol [54] as well as to the presence of ovulatory follicles in the ovaries [52]. In fact, the presence of tertiary follicles in the ovaries are responsible for the elevated 17β-oestradiol plasma concentrations, which in turn induce physiological changes in the reproductive organs, such as hyperemia at the vulvar level and act on the hypothalamus which induces the behavioral estrus signals that lead to the acceptance of the male [55]. Moreover, it is quite well-defined that the color of the vulva at the AI greatly influences fertility [56] and ovulation frequency [40]. In our study, although the receptivity and 17β-oestradiol plasma levels showed differences between the groups, they are not correlated with an increase in fertility. The lack of significance for the fertility of the rabbit does, however, could also be affected by the high sensitivity of the chi-square test comparative to the sample size. It was reported that *Lyceum barbarum* polysaccharides were able to restore the production of sex steroids in the ovaries of female senile rats [41]. Moreover, Liu et al. [40] found that *Lycium barbarum* polysaccharide improved the ratio between the different types of follicles, increased the litter size, weaning survival, and hormonal secretion, and reduced the damage of the ovary during repeated superovulation in mice. It was also reported that the administration of an extract of *Lycium chinense Miller* induced ovulation in adult female rabbits [57]. Based on our findings, goji berry seems to be able to enhance sexual receptivity and affects the estrogen secretion of rabbit does. It would also be interesting to evaluate the effect of the supplementation of the fruit on the primiparous and lactating does, in which the receptivity and fertility are usually reduced for their higher negative energy balance and the inhibitory effect of prolactin secretion on the hypothalamus. This preliminary result can encourage the research to explore a possible role of goji berry in the preparation of female rabbits to AI, with the aim to reduce the use of exogenous hormones in rabbit farms. Nonetheless, further investigation is required to confirm these results and to establish the mechanism of action of the goji fruit, given the scarce literature on this topic. 

To our knowledge, this is the first report that describes the effect of goji berry on the pattern of LH secretion in rabbits. In the present study, goji berry seems to affect the gonadal axis activity by modifying the LH secretion and the estrogenic activity of the ovary. The rabbit is an induced ovulatory species, in which ovulation is induced by the neuro-endocrine reflex. The penile intromission, mounting, and pheromones induce the release of GnRH in a higher pulse-frequency from the medial basal hypothalamus that acts on the GnRH receptors on the gonadotropic cells in the anterior pituitary and determines the LH ovulatory peak within 15–75 minutes [58,59]. In our study, the exogenous administration of GnRH bypassed the neuro-endocrine ovulatory reflex, acting directly on the gonadotropic cells triggering LH release. In the present study, the LH ovulatory peak occurred within 60 minutes by the injection of synthetic gonadotropin in all the groups. These findings are similar to those reported by Brecchia et al. [49], that also found similar values of LH plasma concentrations after the induction of the ovulation. With respect to our results, other authors showed differences in the LH plasma levels detected and/or on the time of the peak of the gonadotropin after the injection of the GnRH analogs, probably linked to a different method of assay or to an individual variation among the animals [60,61]. 

In our study, the G1 group tended to differentiate from G3 and C in LH mean concentrations and LH AUC, respectively. The effect of goji berry on the LH concentration has been poorly investigated in humans and animals, in particular in the female. Several studies evaluated the effect of goji berry on the male reproductive tract and hormone concentrations in other species. The administration of goji berry polysaccharides reduced the testis spermatic injury induced by Bisphenol A and significantly increased the LH plasma levels of male mice [62]. Another study showed that *Lycium barbarum* polysaccharides exhibited a protective effect on fertility and reproductive hormone secretion impairments by activating the gonadal axis in streptozotocin-induced type-1 diabetic male mice [63]. Goji berry polysaccharides had protective effects against damage to the testicular tissue, annexed glands, and LH, FSH, and testosterone secretion of normal and hemicastrated male rats induced by heat exposure (43 °C), and on the DNA damage to mouse testicular cells induced by hydrogen peroxide [64]. Recent research showed that dietary integration with goji berry had positive effects on boar semen quality; in particular, it improved progressive motility, total abnormality rate, sperm concentration, and total sperm per ejaculate compared to the control group [65]. Moreover, the wolfberry group showed a significant reduction of the head, tail, and total abnormality rates in both fresh semen and semen stored for 72 hr in comparison to the control group [65]. Finally, it was reported that goji polysaccharides improved sperm quality and fertility rate after cryopreservation in male Cashmere goats [39]. These findings support the idea that goji berry may affect the reproductive hormonal secretion, and as a consequence, the reproductive performance of the animals, including rabbits. It remains to be established which component could be responsible for the action on the gonadal axis of rabbit does as well as the site and the mechanism of action. An up-regulation of the pituitary receptors and/or increased secretion of GnRH secretory neurons, or the synthesis of LH by the gonadotropic cells of the pituitary, might be speculated. 

In the present research, we found that a low dose of goji berry integration increases milk production and the weight of the litter at birth and weaning. These results are in agreement and confirm the data reported by Menchetti et al. [47], though they used a smaller sample size. Given the missing data on this topic, we can speculate that some components of the *Lycium barbarum* berry may favor the production of milk in different ways, as reported for other nutraceutical substances in other animal species: increasing the concentration of the components and precursors of milk at the level of the mammary gland [66], stimulating the proliferation of the mammary epithelial cells enhancing the uptake of some precursors of milk such as propionate and butyrate [67], enhancing the secretion of some hormones such as prolactin [68], and acting as key regulators of the signal transduction pathways during the synthesis of the components of milk [69]. Goji berry is rich in free amino acids, particularly in L-arginine [70] which is able to increase milk production in cows [67] and sows [68]. 

The energy balance of the does in commercial rabbit farms is generally critical. In fact, females are quite often inseminated during lactation when a high energy output is present because of the milk secretion. The loss of energy is not completely compensated for by feed intake, and consequently, the does increase the mobilization of fat reserves and lose energy which negatively affects the reproductive activity [71]. Several metabolic hormones and metabolites are involved in maintaining energy homeostasis during both pregnancy and lactation in rabbit does [26,27]. These hormones and metabolites acting by complex interactions can influence the ovarian axis hormonal secretion, and as a consequence, they play a role in the relationship between energy balance and reproductive efficiency [27]. Moreover, the development of the mammary gland and milk secretion is under hormonal control in adult rabbits [72]. Milk production is influenced mostly by the reproductive (estrogens, progesterone, and prolactin) and metabolic (GH, corticosteroids, thyroid hormones, and insulin) hormones [73]. It was shown that goji berry affects the secretion pattern of metabolic signals such as leptin, insulin, NEFA, and glucose, improving energy homeostasis [22]. Moreover, *Lycium barbarum* has therapeutic effects in glucosides of Tripterygium wilfordii Hook f (GTW)-induced dyszoospermia rats, improving the semen quality and positively affecting the secretion of reproductive hormones, including prolactin [74]. Consequently, it might be suggested that the higher milk yield in the group supplemented with 1% of goji compared to control does in our study can be due to the hormonal framework of both reproductive and metabolic hormone levels. 

Our findings showed that the does fed with goji berry had a higher litter size at weaning. Other authors found that a low percentage of goji berry induced lower pre-weaning mortality and, at the same time, higher litter size and weight at the opening of the nest (day 18) and at weaning (day 35) compared to animals fed without goji berry supplement [47]. In the same study, the rabbits that received a diet integrated with goji berry exhibited not only a higher mean body weight during the fattening period and at slaughter, but also a better feed conversion rate compared to control animals. The higher quantity of milk ingested by the litters belonging to the goji groups is probably responsible for the greater productive performance. In fact, a link between the quantity and quality of milk produced by the female and the growth performance of the litter, at least until the weaning was reported [68]. It should be noted that rabbits supplemented with the highest dose of goji berry (i.e., 3%) showed no relevant effects compared to the control group; however, they showed minor changes compared to those obtained with the lowest dose. Then, a non-linear dose-dependent effect was found confirming that the use of high percentages of goji requires further investigation. It has been shown, for example, that substances contained in the *Lycium barbarum,* such as polyphenols, may have negative effects when they reach high levels or are administered for long periods, altering the hormonal and/or energy balance in both male and female farm animals [47,75]. In particular, polyphenols could have estrogen-agonistic or antagonistic effects and could interfere with reproductive functions at different levels of the gonadal axis [75]. However, the effects of polyphenols could be species-specific and depend on the polyphenol profile of each plant [75]. 

The effects of goji berry dietary supplementation in livestock animals are limited, but there is evidence that the fruit can enhance growth performance in piglets [76], broiler chickens [20], and hybrid groupers [77]. It was reported that some components of the fruit can enhance the intestinal absorption of nutrients [78] and show antioxidant and immunomodulatory actions [79]. There is evidence that *Lycium barbarum* induces changes in the intestinal microbiota, which in turn, can favor the digestion and the absorption of the nutrients [20,80]. Moreover, goji berry polysaccharides can stimulate the gut immune system, increasing the defense of the animals against pathogen infection through direct and indirect actions such as competition for common nutrients and niches and the increase of host defense, respectively [20,75]. Polysaccharides, acting as a prebiotic, favor the growth of beneficial bacteria, which in turn stimulate the immune system and in particular, the innate immune response [81]. It is possible to suggest that the different compounds of goji berry included in the milk and in the feed may stimulate the growth of the animals, influencing the digestion and absorption of the feed by action on the intestinal microbial population. Moreover, the microbiota affecting the development, maturation, and response of the immune system, may contribute to reducing the infectious disease and the mortality of the rabbit. Taken together, these mechanisms can explain the positive effects of goji berry on the growth performance, and more in general, may contribute to the maintenance of good health status and animal welfare. 

## 5. Conclusions

In conclusion, the present study provided experimental support for the effects of goji berry on the hormonal profile as well as the reproductive and productive performance of rabbit does. In particular, 1% goji supplementation influences the hormonal pattern and increases both receptivity and milk production of the does, as well as the growth of young rabbits, although the effects on fertility are limited. 

Therefore, the use of this natural and nutraceutical product could represent a new strategy to reduce the use of drugs in animal farming. At the same time, it could represent an added value, not only for animal welfare as well as their productive and reproductive performance but also for the rabbit industry and breeding. Although these preliminary findings are encouraging and suggest a potential use of the berries on rabbit nutrition, further studies with a larger number of animals are required to definitely establish both the efficacy and the mechanism of action of the goji berry to improve the reproductive performance of rabbit does, as well as to make a cost-analysis to assess the sustainability of its use in rabbit breeding. In addition, the dose-effect of goji berries could be very interesting for further research.

## Figures and Tables

**Table 1:** Formulation and chemical composition (as fed) of control (C) and experimental diets supplemented with 1% (G1) and 3% (G3) Goji berries.

Ingredients/Analytical Data	Diet		
	C	G1	G3
**Ingredients** ^1^			
Wheat bran	30.0	29.5	29.0
Dehydrated alfalfa meal	42.0	41.5	41.0
Barley	9.5	9.5	9.0
Sunflower meal	4.5	4.5	4.2
Rice bran	4.0	4.0	3.9
Soybean meal	4.0	4.0	3.9
Calcium carbonate	2.2	2.2	2.2
Cane molasses	2.0	2.0	2.0
Dicalcium phosphate	0.7	0.7	0.7
Vitamin-mineral premix ^2^	0.4	0.4	0.4
Soybean oil	0.4	0.4	0.4
Salt	0.3	0.3	0.3
Goji berries	-	1.0	3.0
**Analytical data** ^1^			
Crude Protein	15.74	15.64	15.66
Ether extract	2.25	2.23	2.47
Ash	9.28	9.36	9.25
Starch	16.86	17.07	16.99
NDF	38.05	38.55	37.49
ADF	19.54	19.60	19.01
ADL	4.01	4.31	3.98
Digestible Energy ^3^	2464	2463	2459

^1^ as percentage (%). ^2^ Per kg diet: vitamin A 11,000 IU; vitamin D3 2000 IU; vitamin B1 2.5 mg; vitamin B2 4 mg; vitamin B6 1.25 mg; vitamin B12 0.01 mg; alpha-tocopherol acetate 50 mg; biotine 0.06 mg; vitamin K 2.5 mg; niacin 15 mg; folic acid 0.30 mg; D-pantothenic acid 10 mg; choline 600 mg; Mn 60 mg; Fe 50 mg; Zn 15 mg; I 0.5 mg; Co 0.5 mg. ^3^ as Kcal/kg. Estimated by Maertens et al. [45].

**Table 2:** Productive performance of the control group (C) and does supplemented with 1% (G1) or 3% (G3) of Goji (*n* = 25 does/group).

Parameter	C	G1	G3	RMSE	*p* Value
Pre-weaning mortality (%)	25.1	16.7	22.8	16.1	0.176
Litter size at birth (*n*)	6.5	7.2	6.4	1.9	0.249
Litter weight at birth (g)	339 ^a^	408 ^b^	356 ^ab^	80	0.008
Litter size at weaning (*n*)	4.8 ^a^	6.0 ^b^	4.9 ^ab^	1.6	0.020
Litter weight at weaning (g)	3634 ^a^	5579 ^b^	4966 ^b^	1255	<0.001

RMSE: root-mean-square error. a: b: Means sharing the same superscript are not significantly different from each other (*p* < 0.05).

## Data Availability

The data presented in this study are available on request from the corresponding author.
